# Multidrug-resistant tuberculosis in Ethiopia: efforts to expand diagnostic services, treatment and care

**DOI:** 10.1186/2047-2994-3-31

**Published:** 2014-10-03

**Authors:** Fantahun Biadglegne, Ulrich Sack, Arne C Rodloff

**Affiliations:** College of Medicine and Health Sciences, Bahir Dar University, Bahir Dar, Ethiopia; Institute of Medical Microbiology and Epidemiology of Infectious Diseases, University Hospital, University of Leipzig, Leipzig, Germany; Institute of Clinical Immunology, University Hospital, University of Leipzig, Leipzig, Germany; Translational Centre for Regenerative Medicine (TRM)-Leipzig, University of Leipzig, Leipzig, Germany

**Keywords:** MDR-TB, *M. tuberculosis*, Risk factors, Ethiopia

## Abstract

The emergence of drug-resistant tuberculosis (TB), particularly multidrug-resistant (MDR) and extensively drug-resistant (XDR) TB, is a major public health problem. The purpose of this review is to describe the current status of MDR-TB and factors that increase the risk of this infection. We conducted a systematic review of the literature on MDR-TB in Ethiopia. Out of 766 articles, 23 were found to meet eligibility criteria and included in this review. Among the 23 papers, six of them reported high prevalence of MDR-TB in the range of 3.3%-46.3%. Likewise, two studies reported XDR-TB in the range of 1% - 4.4% in Ethiopia. The most powerful predictor of the emergence of MDR-TB reported in Ethiopia is previous exposure to anti-TB drug treatment. This review indicated that MDR-TB in Ethiopia is a serious public health problem that needs to be addressed urgently. Strengthening early case detection and proper treatment of drug-susceptible TB in accordance with World Health Organization (WHO) treatment guidelines to ensure adequate treatment success rates is critical. Consequently, efforts have been made to a rapidly increase MDR-TB diagnosis as well as the number of treatment sites to implement a directly observed treatment, short-course (DOTS) plus strategy to interrupt transmission of MDR-TB.

## Introduction

Tuberculosis (TB) remains a major global public health problem. It causes illness among millions of people each year and is ranked as the second leading cause of death from an infectious disease worldwide [1]. Globally around 8.6 million new TB cases in 2012 were reported and 1.3 million die every year as a result of TB [1]. Ethiopia, having experienced a major increase in the burden of TB, presents one of the most serious public health challenges. Ethiopia is highly afflicted by the TB pandemic and is ranked second after Nigeria in Africa and seventh among the 22 high TB burden countries worldwide [2].

Multidrug-resistant (MDR) TB has become a major public health problem and presents new barriers to the control of TB [1]. Drug-resistant TB is a man-made problem, largely being the consequence of human error as a result of poor supply management and quality of anti-TB drugs and inadequate or improper treatment, which is further exacerbated by human immunodeficiency virus (HIV) [1]. Poor infection control practice has also been identified as a major contributing factor for the spread of drug-resistant TB [1]. Nearly half a million cases of MDR-TB emerge every year, but only 3% of them get treatment globally and 110,000 die annually [3]. The World Health Organization’s (WHO’s) 2010 Global MDR-TB report estimated that there were 440,000 MDR-TB cases [3.6% (95% CI: 3.0-4.4)] and 150,000 deaths due to MDR-TB worldwide in 2008 [4]. China and India accounted more than half of the MDR-TB worldwide [4]. According to a WHO/IUATLD (World Health Organization/International Union Against Tuberculosis and Lung Disease) survey of 20 countries with the highest rates of MDR-TB among previously treated cases, 14 were in the European Region [5]. In Africa 69,000 MDR-TB cases were reported in 2008 [4]. Extensively drug-resistant TB (XDR-TB) has been reported worldwide and an estimated 9.6% (95% CI: 8.1%-11%) of MDR-TB cases are XDR-TB [1].

Ethiopia is one of the 27 high MDR-TB countries; it is ranked 15^th^ with more than 5000 estimated MDR-TB patients each year [3]. According to the WHO report, the prevalence of MDR-TB has been 2.8% in newly diagnosed patients; it is reportedly even higher in patients who have previously received anti-TB treatment 21% [1, 6]. Published studies on MDR-TB are increasingly available worldwide, but accurate data on drug-resistant TB in Ethiopia is limited. Upon this background, this review provides a comprehensive and up-to-date assessment of the status of the MDR-TB epidemic in Ethiopia, following up on a series of reports on anti-TB drug-resistance previously reported by WHO. Therefore, the purpose of this review is to describe the current status of MDR-TB in Ethiopia, the factors that increased the risk of drug resistance and discuss the importance of our findings for informing TB control strategies in Ethiopia.

## Methods

The literature search strategy in this paper included searching PubMed/Medline and the Google scholar database using keywords such as “Tuberculosis” and “MDR-TB”, “XDR-TB”, “risk factors”, “drug resistance”, “incidence rate”, and “control”. Each term was searched separately with the name of the study region. Only English language papers and WHO websites were included in the search and the searches were focused on studies of prevalence, drug resistance, MDR-TB, XDR-TB and major risk factors associated with drug-resistant TB. Literature that did not report on a study of MDR and XDR-TB were excluded. The three authors independently reviewed all of the studies found and WHO websites. A structured form for data extraction was used and included such points as the place where the study was conducted, year of publication, TB incidence rate, prevalence of any drug resistance, isoniazid (INH) and rifampicin (RMP) resistance, MDR- and XDR-TB, risk factor for the incidence and drug-resistant TB. The frequency of prevalence, drug resistance of TB and risk factors reported in all the selected studies were assessed and tallied. In this review we report the risk factors most commonly reported in the literature. Meta-analysis was not used to assess risk factors due to differences in the methodologies used and incomplete result reporting.

### Operational definitions

The following definitions relating to drug resistance were used [4, 7, 8]:

-Drug-resistant TB - TB that is resistant to any first-line anti-tuberculosis drug.
-Isoniazed monoresistant TB - TB caused by strains of *M.tuberculosis* that are resistant to only INH.
-Rifampicin monoresistant TB - TB caused by strains of *M.tuberculosis* that are resistant to only RMP.
-MDR-TB – TB caused by strains of *M.tuberculosis* that are resistant to at least INH and RMP.
-XDR-TB - MDR-TB plus resistant to fluoroquinolone and at least one second-line injectable agent: amikacin, kanamycin, and/or capreomycin
-Primary drug resistance - Drug-resistant TB in a person with no history of TB treatment, implying they were infected with a resistant TB. This reflects person-to-person transmission of drug-resistant TB bacilli.
-Acquired drug resistance - Drug-resistant TB in a person with a history of TB treatment. This reflects drug resistance acquired during TB treatment but may also reflect infection or re- infection with resistant TB bacilli.

## Review

### Findings

**Figure 1:**
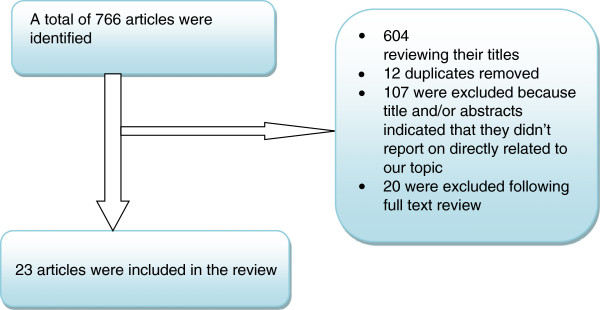
**Flow diagram that shows literature review.**

**Figure 2:**
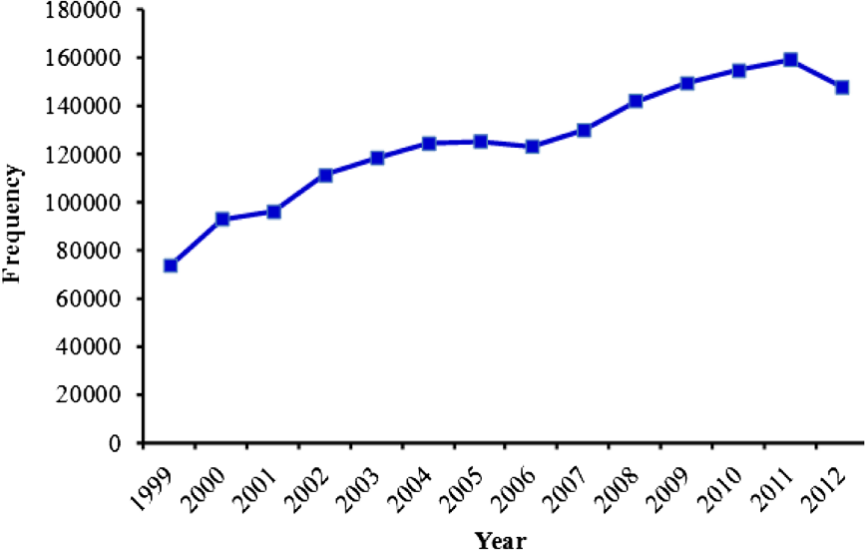
**TB case notification rate in Ethiopia,** **1999–** **2012.** Source: WHO report 2013 global TB control.

**Figure 3:**
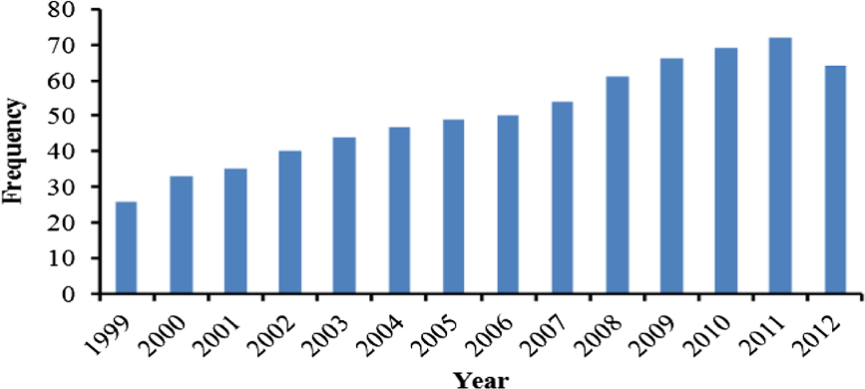
**Case detection rate of TB in Ethiopia,** **1999–** **2012.** Source: WHO report 2013 global TB control.

**Figure 4:**
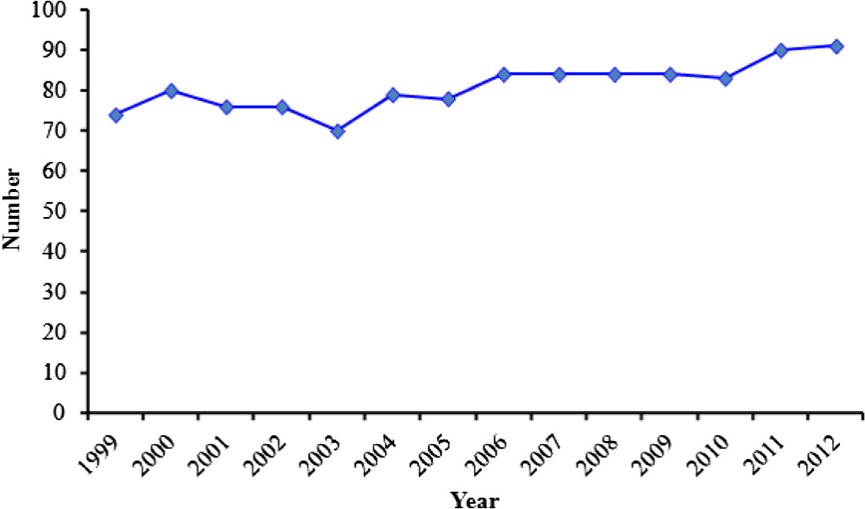
**Treatment success rate of TB in Ethiopia,** **1999–** **2012.** Source: WHO report 2013 global TB control.

**Figure 5:**
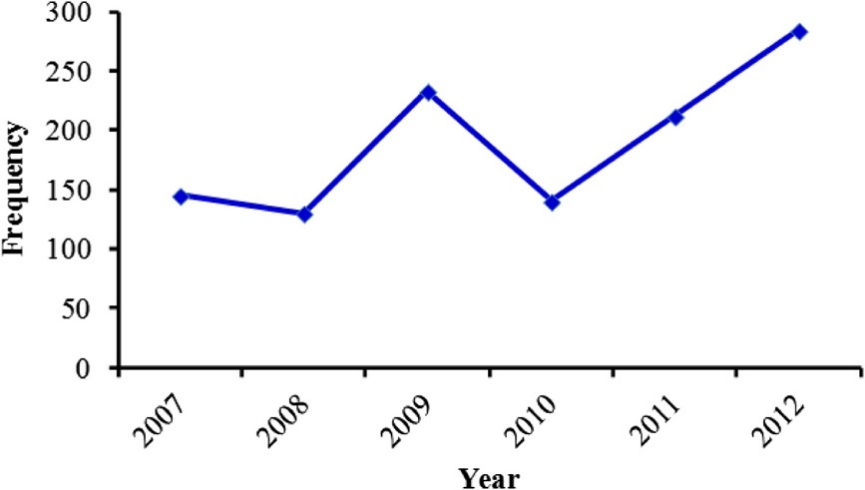
**Proportion of MDR-** **TB in Ethiopia,** **2007–** **2012.** Source: WHO report 2013 global TB control.

**Figure 6:**
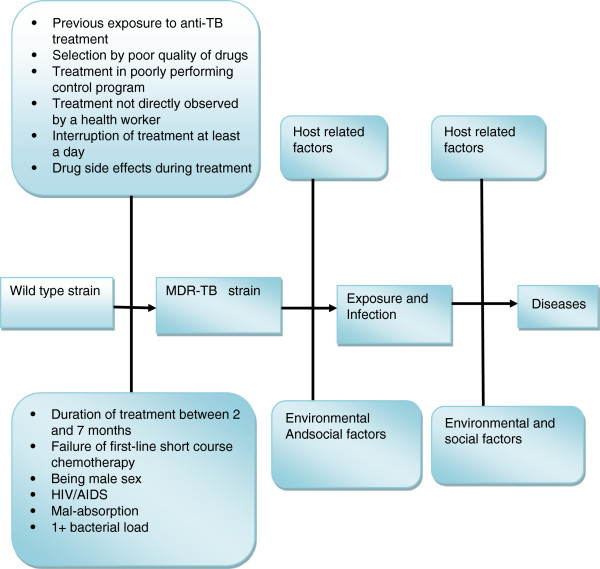
**Risk factors associated to the occurrence of MDR-** **TB in Ethiopia**[19, 21–24, 27, 31–33]. Adapted from Ref. No. 7.

Our electronic search resulted in 766 citations. Out of 766 articles, 604 were excluded after reviewing their titles, 12 were found to be duplicates, 107 were excluded because the title and/or abstracts indicated that they did not report on subjects directly related to our topic and 20 were excluded following full text review. A total of 23 papers were met our eligibility criteria and were included in the review (Figure 1). Among the 23 papers, six of them reported high prevalence of MDR-TB in the range of 3.3%-46.3%. Likewise, two studies reported XDR-TB in the range of 1% - 4.4% in Ethiopia (Table 1). Ethiopia’s TB case notification, case detection and treatment success rate are summarized in Figures 2, 3, and 4, respectively. Trends in the MDR-TB detection rate among all cases from 2007 to 2012 showed an increase from 130 cases to 284 cases (Figure 5). Figure 6 presents a summary of risk factors associated to the occurrence of MDR-TB in Ethiopia. HIV/AIDS, previous exposure to TB treatment, exposure to a known MDR-TB case, history of using poor quality TB drugs, treatment in a poorly-performing control program, mal-absorption, treatment not directly observed by a health worker, being male, and failure of first-line short-course chemotherapy were found to be associated with increased risk of MDR-TB in Ethiopia. Previous exposure to anti-TB drug treatment was found to be the most powerful predictor of MDR-TB incidence reported, followed by HIV/AIDS, in Ethiopia (Table 2).

**Table 1:** **Reported drug**-**resistant TB in Ethiopia**, **1994**-**2012**

Author	Study time	Any drug resistance%	INH%	RMP%	MDR- TB%	XDR- TB%
Demissie et al. [12]	1994	15.6	8.4	1.8	1.2	NR
Abate et al. [13]	1998	50	45	12	12	NR
Bruchfed et al. [14]	1996-1997	14	8.3	2.5	0.8	NR
Gebeyehu et al. [15]	2001	19.5	7.6	0	0	NR
Desta et al. [16]	2004-2005	27.4	5.5	1.4	0	NR
Asmamaw et al. [17]	2004-2005	21.4	13.3	1.2	NR	NR
Wright et al. [18]	2002-2007	26.9	7.7	2.7	1.6	NR
Agonafir et al. [19]	2005-2006	60.8	54.2	43.9	43	4.4
Hussen et al. [20]	2011	36.3	29.4	13.7	11.8	1
Yimer et al. [21]	2008	30.1	3.2	1	1	NR
Abate et al. [22]	2004-2008	72.9	56.1	46.5	46.3	NR
Tessema et al. [23]	2009	15.8	13.8	5.8	5	NR
Abebe et al. [24]	2010-2011	18.4	13.2	2.2	1.5	NR
[Internet] [25]	2010-2011	31	26	12	9.7	NR
Biadglegne et al. [26]	2012	6.7	3.6	1.8	1.3	NR
Esmael et al. [27]	2010-2011	33.5	5.2	0.9	6.5	NR
Ejigu et al. [28]	2005	0.0	55.2	32.8	32.8	NR
Demissie et al. [29]	1998	12.9	8.4	0.6	0.6	NR
Wolde et al. [30]	1986	15.2	12.0	1.1	3.3	NR

*NR*- not reported.

**Table 2:** **Positive vs negative association of risk factors with MDR**-**TB in Ethiopia**

Risk factor	Positive association with drug resistance	Negative association with drug resistance
Previous exposure to anti-TB treatment	Agonafir et al. [19], Abate et al. [22], Tessema et al. [23], Esmael et al. [27], Hirpa et al. [31], Berhan et al. [33]	Biadglegne et al. [26]
1+ bacterial load	Esmael et al. [27]	
Drug side effects to first-line anti-TB drugs	Hirpa et al. [31]	
Treatment not directly observed by a health worker	Hirpa et al. [31]	
Interruption of treatment at least a day	Hirpa et al. [31]	
HIV/AIDS	Yimer et al. [21], Abebe et al. [24]	Abate et al. [22], Tessema et al. [23], Hirpa et al. [31], Berhan et al. [33]
Age		Yimer et al. [21], Abate et al. [22], Tessema et al. [23], Esmael et al. [27], Hirpa et al. [31], Berhan et al. [33]
Being male	Biadglegne et al. [26]	Yimer et al. [21], Abate et al. [22], Tessema et al. [23], Esmael et al. [27], Hirpa et al. [31],
Newly treated cases	Biadglegne et al. [26]	Agonafir et al. [19], Abate et al. [22], Tessema et al. [23], Esmael et al. [27], Hirpa et al. [31], Berhan et al. [33]
Treatment in poorly performing control program	FMOH [32]	
Exposure to a known MDR-TB case	FMOH [32]	
History of using poor quality of drugs	FMOH [32]	
Mal-absorption	FMOH [32]	
Failure of first-line short-course chemotherapy	FMOH [32]	

## Review

### Epidemiological evidence of MDR-TB in Ethiopia

The WHO report in 2013 indicated that 450,000 new cases of MDR-TB and 170,000 deaths due to MDR-TB occurred globally in 2012. In Africa data on MDR-TB is scarce. However, between 2007 and 2012, a total of 65,422 MDR-TB cases were reported by 15 countries [1, 9]. South Africa comprises of 87.9% of the African burden of MDR-TB [1]. The current WHO estimate of MDR-TB prevalence among all cases in South Africa is 15,419. Likewise, 2336 XDR-TB cases were reported from five countries [9], of these 97.6% of them were in South Africa. South Africa remains the country that reports the highest XDR-TB cases in the world and the annual notification rate increased from 467 in 2009 to 1596 in 2012 [1]. About 10% of MDR-TB cases reported in this country were XDR-TB. Genotype studies have shown that between 63% and 75% of XDR-TB cases progress through acquisition of resistance [10].

In Ethiopia, the incidence of MDR-TB strains is a continuing challenge to the TB control program. The first national surveillance data during the 2003–2005 study periods reported 1.5% MDR-TB in Ethiopia [11]. Ethiopia has been one of the highest TB burden countries with respective incidence and prevalence rates of 247 and 470 cases per 100,000 in 2012 [1]. The value for the estimated number of TB cases in Ethiopia has varied between 431 in 1998 and 247 in 2012 and its highest TB case detection rate and TB treatment success rate was observed in 2011. Likewise, TB case notifications also increased over the years and reached their highest level in 2011. The reason for the increased number of notified cases might be due to the expansion of health care services in the country. The appearance and spread of drug-resistant TB strains in new and previously treated cases worsens the TB problem in Ethiopia [2]. In Ethiopia, a total of 1144 MDR-TB cases were reported between 2007 and 2012 [1, 9]. After falling from 145 to 130 in 2008, it rose to 284 in 2012, indicating a proportional increase of MDR-TB with time. According to our review, the proportion of MDR-TB among all TB cases varies from place to place [12–30]. In a study conducted in the capital of the country, Addis Ababa reported 12% MDR-TB cases [13]. Agonafir *et al*. in 2010 reported 43% MDR-TB and 4.4% XDR-TB. Another study in three sites of Bahir Dar (northwest Ethiopia) indicated 11.8% MDR-TB and 1% XDR-TB cases [20]. Additionally, 46.3% of MDR-TB was reported by Abate *et al*. in 2012 and 9.7% by Ali *et al*. in the same year among all cases. Another report on northwest Ethiopia showed a higher rate at 5% of MDR-TB patients [23]. Studies in other parts of the country indicated 1.3%, 6.5% and 3.3% of MDR-TB [26, 27, 30]. Ejigu *et al*. in 2008 [28] and Hirpa *et al*. in 2013 [31] reported 32.8% and 134 MDR-TB cases, respectively. Reports in other countries were consistent with this overall trend [1, 8, 9, 18]. MDR-TB and XDR-TB incidence in all of the reported studies indicate the spread of MDR-TB and XDR-TB strains and that the local control measures for the prevention of this deadly disease are unsatisfactory. This suggests that establishing diagnostic facilities for early case detection and treatment of MDR-TB in the country is significant.

### Risk factors

The review indicated that previous exposure to TB treatment was found to be the most significant risk factor reported in Ethiopia [19, 22, 23, 27, 31–33]. Similar findings were reported from studies conducted in Europe, India, China, Portugal, Iran, Spain and east Africa [34–41]. In contrast to this report Biadglegne et al. in 2013 [26] reported that newly treated TB cases harbor MDR-TB in Ethiopia. Yimer et al. in 2011 [21] and Abebe et al. in 2012 [24] reported HIV to be a risk factor for MDR-TB. However, a positive association between HIV and MDR-TB has not been reported from study results in east Africa and Ethiopia [22, 23, 31, 33, 41]. According to the Federal Ministry of Health in Ethiopia [32], exposure to a known MDR-TB case, history of using poor quality TB drugs, treatment in a poorly-performing control program and mal-absorption were found to have a positive association with MDR-TB in Ethiopia. A case control study in Addis Ababa, Ethiopia by Hirpa et al. in 2013 [31] reported drug side effects during treatment (OR., 4.5, 95% CI., 1.9-10.5), treatment not directly observed by a health worker (OR., 11.7%., 95% CI., 4-34.3), interruption of treatment of at least a day (OR., 13.1., 95% CI., 3.0-56.6), duration of treatment between 2 and 7 months (OR., 14.8., 95% CI., 2.3-96.4), and treatment with a category II regimen (p = 0.000) as risk factors for MDR-TB. Being male has been reported to be a risk factor for MDR-TB [22, 26]. However, these reports contradict the reports for other studies in the county [19, 22, 23, 27, 31, 33]. The design of the studies, factors such as sample size, education, place, study subjects and other factors might be reasons for the discrepancies in determining the risk factors associated to MDR-TB.

The WHO report in 2013 also confirmed that the highest prevalence of MDR-TB among new and previously treated TB cases was 3.6% and 20.2%, respectively, which is more than 17 times higher in previously treated patients. In contrast to the results of this review, Antunes *et al*. in 2000 [37] reported that social factors such as drug abuse, poverty, and homelessness induces treatment failure and facilitates the emergence of MDR-TB. Alcoholism and diabetes were also reported from Spain as important predictors of MDR-TB [42]. Overall, previous exposure to TB treatment was found to be the most frequently reported factor of increased risk of MDR-TB in Ethiopia. The high association of previous TB treatment to MDR-TB might be explained due to inappropriate chemotherapy regimens, inadequate or irregular drug supply, unsatisfactory patient or clinician compliance, lack of supervision of treatment and absence of infection control measures in hospitals [37].

### TB treatment regimens

Chemotherapy regimens that are used for the treatment of all types of TB are classified as first- and second-line anti-TB drugs [1]. First-line anti-TB drugs include isoniazid (INH), rifampicin (RMP), pyrazinamide (PZA), ethambutol (EMB) and streptomycin (STM). INH and RMP are the two most commonly used drugs for treatment of TB. First-line anti-TB drugs are safe and effective if used correctly [1]. The effective treatment of MDR-TB is critical to reducing the spread of drug-resistant TB in the community. Drug groups for the treatment of MDR-TB and XDR-TB treatment regimens have been reviewed recently [43] and will not be covered here. Currently, TB care and treatment has become more complicated due to the emergence of M/XDR-TB. Second-line drugs that are used for the treatment of MDR-TB are listed as aminoglycosides; e.g., amikacin (Am) and Kanamycin (Km); polypeptides: e.g., capreomycin (Cm), viomycin and enviomycin; fluoroquinolones; e.g., ciprofloxacin (Cip), levofloxacin (Lfx), ofloxacin (Ofx), moxifloxacin (Mxf) and gatifloxacin; and thioamides: e.g., ethionamide (Eto), prothionamide and cycloserine (Cs), and P-aminosalicylicacid (PAS) [1]. Second-line anti-TB drugs are less potent, need to be administered for a much longer time, are more toxic and are high-cost compared to first-line anti-TB drugs [43]. Agents with unclear roles in drug-resistant TB treatment are called third-line anti-TB drugs such as clofazimine (Cfz), linezolid (Lzd), amoxicillin/clavulanate (Amx/Clv), thioacetazone (Thz), imipenem/cilastatin (Ipm/Cln) and high-dose isoniazid [1].

### MDR-TB treatment regimens in Ethiopia

Obviously, the treatment of MDR-TB requires a longer duration, is considerably more complicated, expensive and toxic [1]. The longer treatment course of MDR-TB results in poor treatment outcome, leading to the emergence of XDR-TB. XDR-TB treatment is much more difficult and costly and will stress national health budgets even more than MDR-TB treatment [4]. The first patients were admitted for MDR-TB treatment in Ethiopia in 2009 in a rehabilitated isolation ward in St. Peter’s Hospital in Addis Ababa [3]. In the same year, 45 MDR-TB patients were enrolled initially in the second phase at St. Peter’s Hospital. However, there has been a rapid scale-up of drug-resistant TB care in the last five years; in 2014, at the national and regional state level, there were 19 care sites for drug-resistant cases and therapy was initiated on 811 patients in 2012. Of these nine of them were XDR-TB. Progress is being made; however, the response is too slow given the prevalence MDR-TB in Ethiopia. This is because the expanding access to care for MDR-TB cases is limited to the main and regional large cities. As a result of this, MDR-TB patients in rural and remote areas may not have access to health care services, may prefer consultation with traditional healers that are more readily available and come late to health care centers (personal communications). Delayed case detection and treatment of MDR-TB cases might also contribute to the spread of the disease in the community [32]. Thus, there is a need to train health extension workers and volunteers how to screen and care for MDR-TB cases at the community level. Continuous public health awareness about MDR-TB at the community level is also very important to reduce the spread of this deadly disease. The facilities that are equipped to treat MDR-TB are supported by the Ministry of Health and non-governmental organizations (NGOs). The treatment policy for MDR-TB in Ethiopia combines standardized and individualized treatment based on second-line drug susceptibility testing [3]. A standard regimen is given to all MDR-confirmed cases daily under direct observation by a health care worker at a health care centre and by family DOTS supporter (s) at home [3]. Furthermore, continuous monitoring and building capacity for family DOTS supporters are essential components of the DOTS strategy. The regimens include at least four drugs that are certain or expected to be effective and the duration is a minimum of 18 months after culture conversion [3]. The purpose of effective treatment of drug susceptible TB is curing the patient, interrupting transmission of TB to other persons, and preventing the development of drug resistant strains [1]. These goals are not being achieved in many regions of the country though anti-tuberculosis drugs are available. This might be due to either patient non-adherence to treatment or clinicians’ non-adherence to the national treatment guidelines or both [44].

### Mechanisms of drug resistance in *M.tuberculosis*

Understanding the mechanism of drug resistance to anti-TB drugs not only helps to put measures into practice to limit the spread of such resistance but also enables us to recognize genes linked with drug resistance [7]. Most chromosomal mutations in the genome of *M.tuberculosis* that confer resistance to anti-TB drugs occurred spontaneously [45]. Data from the Johnson et al. report in 2006 showed the rate of spontaneous mutation for INH and RMP is 3.4×10^-6^ and 3.1×10^-8^, respectively. However, the amplification of the above mentioned genetic mutation results in drug-resistant TB. Mechanisms of drug resistance to first- and second-line drugs have been reviewed recently [7, 45] and will not be reviewed here.

### Diagnosis of drug-resistant TB

Laboratory investigations are a vital part of the clinical assessment and guide the selection of drugs for patient management. One main element of the TB control strategy is effective case management through early detection and treatment of the patients [1], which is economically beneficial since Ethiopia uses expensive drugs as first- and second-line anti-TB treatment. Thus, laboratory availability for performing drug resistance testing is crucial, since diagnosis of drug-resistant TB usually requires pure culture of *M. tuberculosis*. MDR-TB is a particularly threatening infection that is difficult to diagnose without a proper laboratory facility. The most critical factor in addressing MDR-TB in Ethiopia is the lack of laboratory infrastructure and transport networks that can provide rapid diagnosis. In addition, transmission dynamics of MDR-TB in Ethiopia are not well understood. In fact, laboratory services in Ethiopia remain in a stage of fine needle aspiration cytology (FNAC) and Ziehl Nielsen (ZN) smear in clinical specimens [46]. FNAC and ZN smear lack sensitivity and specificity. Moreover, these methods are limited due to lack of species identification and drug susceptibility testing. Culture-based drug susceptibility testing methods can provide definitive results, but are labor intensive, time consuming [47] and generally unavailable in resource-limited settings where TB is endemic. For instance in Ethiopia, drug susceptibility tests for Mycobacterium species are not available as routine tests, not even for patients with suspected infection by drug resistant strains. So far, there are two laboratories in the capital city Addis Ababa that perform molecular (LPA) and conventional drug susceptibility tests. Due to this problem, empiric treatment for MDR-TB suspects is common, resulting in problematic treatment outcomes of MDR-TB cases.

Molecular methods such as real-time polymerase chain reaction (RT-PCR), solid phase hybridization assays and sequencing that target drug resistance mutations are suitable approaches for rapid drug susceptibility testing [48]. Line probe assays (LPAs) such as Genotype MTBDR, the new GenoType MTBDRplus and GenoType MTBDRsl assay (Hain Life Science GmbH, Nehren, Germany) have also been described for detection of MTBC-specific DNA and gene mutation linked with drug resistance from clinical specimens and culture isolates [23, 26, 40, 48–50]. The GenoType MTBDRplus assay detects the resistance to both INH and RMP simultaneously and thus can detect MDR-TB [23, 26]. The GenoType MTBDRsl assay for detection of MTBC resistant to second-line drugs was reported on recently [23]. A real-time PCR based approach, Xpert MTB/RIF assay (Cepheid, Sunnyvale, CA) and a reverse hybridization-based line probe assay, the INNO-LiPA Rif. TB (LiPA) (inogenetics, Ghent, Belgium), have been endorsed by the WHO to rapidly identify *M.tuberculosis* and associated mutations to RMP resistance. The sensitivity and specificity of molecular techniques compared to conventional methods of drug resistance testing was reported [23, 26, 49–55]. In recent years the microscopic observation drug susceptibility (MODS) assay was introduced for detecting drug resistant *M. tuberculosis* from liquid media [56, 57]. Resistance is detected by the presence of anti-tuberculosis drugs and it returns results within seven days [56]. The sensitivity to detect MDR-TB compared to conventional drug susceptibility tests was reported [56, 57].

### Control of MDR-TB in Ethiopia

Infection control is a key element to prevent transmission of MDR-TB. Administrative, environmental and personal respiratory protection measures are key interventions [3]. Good administrative control is the most important in infection control of MDR-TB. In particular, environmental controls and personal respiratory protection measures will not work in the absence of good administrative control measures [3]. These measures must be ensured in all health facilities involved in MDR-TB care in Ethiopia. Effective treatment, control and prevention of emergence and transmission of drug-resistant TB at the community level are required to reduce the increasing trend in MDR-TB in Ethiopia [32]. Environmental control measures should never replace administrative controls; however, they are intended to reduce the concentration of infectious droplet nuclei in the air. Such measures include maximizing natural ventilation and controlling the direction of airflow [3]. Opening windows to increase natural ventilation and the use of fans to control the direction of airflow is crucial to reduce infection in resource-limited settings. Personal respiratory protection protects health-care workers and other people from being infected [1]. The national guideline for control of drug-resistant TB is understood and, theoretically, should be effortless to implement in the country. In contradiction, however, the prevalence of MDR-TB is often increasing in Ethiopia. Clearly a directly observed treatment, short-course (DOTS) strategy for patients is the best weapon against MDR-TB [58] and it comprises political commitment with increased and sustained budgeting, a standardized treatment regimen directly observed by a health care worker, an effective drug supply and management system, case detection through quality assured laboratories, and a monitoring and evaluation system with TB infection control impact measures. The strategy also includes measures to address TB infection control advocacy and to support operational research [58]. The enhanced DOTS program, DOTS-plus has been developed for managing MDR-TB in resource-limited countries and this program recommends additional investment in facilities for culture and drug susceptibility testing for the detection of drug-resistant TB and provision of appropriate second-line drugs [59].

## Conclusion

This review indicated that MDR-TB in Ethiopia is a serious public health problem that needs to be addressed urgently. Several reports indicated that previous exposure of anti-TB treatment increased the risk of MDR-TB in Ethiopia. Thus, strengthening early case detection and proper treatment of drug susceptible TB according to WHO treatment guidelines to ensure adequate treatment success rates is essential. This will limit the emergence of M/XDR-TB and prevent the spread of the disease. Consequently, a rapid build-up of MDR-TB diagnosis and treatment sites for the implementation of the DOTS-plus strategy is critical to interrupt transmission of this deadly disease in the country.
